# Increase in the Prevalence of Online Pornography Use: Objective Data Analysis from the Period Between 2004 and 2016 in Poland

**DOI:** 10.1007/s10508-021-02090-w

**Published:** 2021-11-08

**Authors:** Karol Lewczuk, Adrian Wójcik, Mateusz Gola

**Affiliations:** 1 Institute of Psychology, Cardinal Stefan Wyszyński University in Warsaw, Warsaw, Poland; 2 Nicolaus Copernicus University, Toruń, Poland; 3 Swartz Center for Computational Neuroscience, Institute for Neural Computation, University of California, San Diego, 9500 Gilman Drive, San Diego, CA 92093-0559 USA; 4 Institute of Psychology, Polish Academy of Science, Warsaw, Poland

**Keywords:** Online pornography, Pornography use, Internet, Hypersexuality, Compulsive sexual behavior

## Abstract

Despite the considerable amount of attention presently devoted to the high accessibility of online pornography, very little formal analyses have been carried out to show how the advent and proliferation of Internet technology has changed the prevalence of pornography use in populations. We conducted a preliminary analysis based on objective website traffic data, representing the changes in the number of (1) Internet users generally and (2) online pornography users specifically, between 2004 and 2016 in Poland. We observed a clear increase in the estimated number of people using online pornography in the analyzed period. The estimated number of general population members viewing pornography on the Internet increased over three times (310%) between October 2004 and October 2016–starting from an estimated 2.76 million in the first period to 8.54 million in the last. At the same time, we did not observe a clear increase in the percentage of Internet users who viewed online pornography in the same time period. Additionally, pornography viewership on the Internet was almost 2 times more prevalent among male (47%) than female Internet users (27%), and most popular in the 18–27 age group. Since our analysis is based on objective data, it does not share the limitations inherent in self-reports. However, our approach also has several important limitations (e.g., the analysis does not include online activity generated on mobile devices and under a private browsing mode); thus, the results should be interpreted with caution.

**Supplementary Information:**

The online version contains supplementary material available at 10.1007/s10508-021-02090-w.

## Introduction

Instantaneous and increasingly easier access to pornography, attributed by many to the development of high-speed, broadband Internet technology, has attracted the attention of the scientific community (e.g., Cooper et al., 1999; Owens et al., 2012), non-governmental organizations, social communities that aim to prevent Internet and pornography addiction (*e.g., NoFap* movement, Sproten, 2016), and even government representatives and policy makers. In recent years, online pornography was recognized with a *public health crisis* status in multiple US states (e.g., Dines, 2016), and initiatives for preventing children’s access to online pornography were established, e.g., in the UK (BBC, 2013) and in Poland (STS, 2017). Although these signs of concern can be provoked by reasons other than the scientific, they may also be at least partly related to a growing amount of research, showing that extensive online pornography use can have a range of negative consequences for psychological health, intimate relationships, social connections and brain function (Gola & Draps, 2018; Gola et al., 2016, 2017; Klucken et al., 2016; Kowalewska et al., 2018; Lewczuk et al., 2017; Love et al., 2015; Potenza et al., 2017; Voon et al., 2014). At the same time, drawing definitive conclusions about the effects of pornography seems premature, as the research field is still in its infancy, and there are multiple examples of research pointing to positive or null effects of pornography viewing for physical, sexual as well as psychological health, sexual satisfaction and intimate relationship satisfaction (e.g., Dwulit & Rzymski, 2019; Grubbs & Gola, 2019; Kohut et al., 2017; Leonhardt et al., 2019; Prause et al., 2015; Vaillancourt-Morel et al., 2019). Despite the considerable attention given to the potential effects of viewing pornography, there is still almost a complete lack of formal analyses of changes in the proliferation of online pornography over the years.

### Estimating Changes in the Prevalence of Online Pornography Use over Time

The results of a survey among a representative sample of Norwegians conducted in 2002 showed that almost 90% of people reported previous contact with pornography. Among them, 87.9% of males and 62.9% of females reported seeing a pornographic magazine within their lifetime; 77.2% of males and 55% of females declared that they had watched a pornographic movie in the past; and only 36.6% of men and 8.9% of women reported ever seeing pornographic content on the Internet. Generally, 78.9% of respondents declared that they had never seen online pornography in any form (Træen et al., 2004). At this time, the Internet did not yet seem to have the prominent and leading role in pornography distribution that we ascribe to it today—the survey results favored pornographic magazines and TV or rental movies as prominent mediums of pornography distribution. It seems that this landscape is now different, but, based on available data, it is surprisingly difficult to make much of an assertion about these differences.

To study the changes in the proliferation of a particular form of pornography distribution over time (e.g., online pornography), repeated measure designs (or similar designs) seem to be the most suitable. However, such studies are incredibly scarce, with—to the best of our knowledge—only one available to date (General Social Survey conducted in the USA; Wright, 2013). In view of this, another potentially useful approach appears to be based on the comparison of results from multiple individual studies estimating pornography use at particular points in time for particular populations, which enables us to obtain a full picture from many fragmentary pieces. However, the results of such studies are inherently difficult to reliably compare, and their results vary considerably. In most cases, it is almost impossible to conclude whether the differences between results should be attributed to actual differences in the proliferation of pornography use or to methodological differences between the studies—thus, this potentially useful approach ends up being almost completely ineffective. The above-mentioned issues are discussed in detail in the next subsections.

#### General Social Survey and Pornography Use

For nearly every year during the past 40 years, General Social Survey (GSS) respondents were asked if they had seen an X-rated movie in the past year (“Have you seen an X-rated movie in the past year?”, Yes/No answer). Based on GSS data, Wright (2013) found that, despite an undeniable increase in the proliferation of Internet pornography and an increase in the number of erotic websites during this time, declared pornography consumption among US males had only slightly increased. For example, 32% of male respondents between the years 1987–1997 declared watching an X-rated movie, and 34% in 1997–2010. On average, with each year, the number of GSS male respondents who reported seeing an X-rated movie during the last year increased by less than 0.3% prior to 1986, and only 0.1% afterward (Wright, 2013). A similar analysis conducted by Price et al. (2016) led to similar broad conclusions, but also hinted that the slightly stronger increase between the estimates obtained for the 1972 cohort and the 1980 cohort (10% increase in pornography use for men, and 7% increase for women) might be connected to the advent and development of the Internet (Price et al., 2016). At the same time, both Wright (2013) and Price and colleagues (2016) concluded that the changes in the prevalence of pornography consumption over time, based on a GSS data analysis, are smaller than expected.

It is worth highlighting that GSS data shows changes in general pornography use, irrespective of the medium of pornography consumption (although it excludes still pictures and erotic stories). It is possible that indices of pornography consumption derived from GSS maintain relative stability over time, because they reflect latent, opposing trends—a growing prevalence of online pornography and a diminishing market share for more traditional forms of pornography distribution. Others argue that GSS results may maintain relative stability, because of the outdated way the question about pornography consumption is operationalized (*Have you seen an X-rated movie in the past year?*). Presently, the term *X-rated movie* does not seem to be used to describe pornographic material as frequently as before and some people may not think that short pornographic clips posted online constitute an *X-rated movie* (Wright, 2013)*.* This directs us to a very important issue—self-report data inherently depends on the individual’s implicit convictions about what constitutes *pornography* or an *X-rated movie*, and opens up a broader discussion on the limitations of using declarative data to estimate the prevalence of pornography use in populations.

### Limitations of Declarative Data for Estimating the Prevalence of Pornography Use

Previous analyses of the prevalence of pornography use in populations are mostly based on self-reported survey data. However, the results of these studies vary greatly, even when only research based on representative populations is considered, as declarative data have a few important limitations which decrease reliability, validity and therefore decrease the utility of obtained results.

Regnerus et al. (2016) carried out a comparative analysis of several fairly recent surveys aimed at estimating pornography use in the US population and showed that estimates of monthly use vary greatly as a consequence of differing methodological approaches. Since there is no unitary methodological standard for measuring pornography use, the rate of pornography consumption in self-report studies is addressed with a palette of various designs (e.g., human vs. computer-assisted interviews, with different levels of influence of social desirability bias), question and answer options and recall period required.

Self-reported pornography consumption measures suffer from different types of errors and biases that can be consequential to the obtained results: social desirability bias, which may cause respondents to underreport activities that violate social norms (Fisher, 1993; Rasmussen et al., 2018); errors related to faulty memory (especially for longer recall periods) or memory bias (e.g., recency bias). As mentioned before, self-report data also depends on individuals’ implicit convictions about what constitutes terms like *pornography*, *erotic material* or an *X-rated movie* (for an extensive discussion of these methodological issues, see: Regnerus et al., 2016; Schroder et al., 2003)*.*

Based on available data, it is hard to estimate the impact that these types of biases may have on respondents’ declarations of pornography consumption. However, we have evidence of the presence and impact of these kinds of biases on self-report measures of other online activities. Junco (2013) reported over a fivefold difference between reported and actual time spent on Facebook during a particular day. Similarly, Kahn et al. (2014) showed that MMO (Massively Multiplayer Online game) game players underreported their playing time when compared with actual time spent on gaming. The correlation between those two measures was positive, but only moderate in size *r* = 0.37. Only 13% of variance in reported time spent on computer games could be predicted by actual time spent gaming (Kahn et al., 2014). Moreover, Auer and Griffiths (2017) showed that online gamblers underestimated their gambling expenditures during a particular month when compared with objective data. In this case, the correlation between both measures was also positive, but also only moderate, *r* = *0.35*, only 12% of shared variance. Moreover, this bias was the strongest for the most active players (Auer & Griffiths, 2017).

As described, evidence indicates that self-report measures can be quite inaccurate when compared with objective measures of behavior. What is more—the examples discussed above refer to domains of behavior that share strong similarities with online pornography consumption. All of them are computer mediated, involve Internet use and for some individuals, they tend to take a compulsive or addictive character (Ashley & Boehlke, 2012; Cash et al., 2012; Gola & Potenza, 2018; Kraus et al., 2018).

Despite the discussed evidence of poor reliability and validity of self-report measures for studying online behavior, previous studies on Internet pornography consumption are almost exclusively based on self-report measures. Supplementing the results obtained through declarative measures with analyses based on objective data seems to, at least in part, address this problem.

## Summary and Aims of the Present Study

In summation, estimating the changes in the prevalence of online pornography in populations on the basis of available data is problematic due to several important factors: (1) There is a lack of longitudinal or repeated measures designs addressing this subject; (2) The General Social Survey, which seems to be the only study providing information about changes in pornography consumption over the years, has important limitations, some of which are inherent to its declarative character; (3) inferring conclusions about longitudinal changes in the prevalence of online pornography on the basis of cross-sectional studies is problematic, as these studies are based on different populations; (4) studies on representative populations are very scarce, as most studies use local samples (e.g., students of a particular university); (5) there is no methodological standard shared by the studies. When operationalizing pornography consumption, individual studies use different wording for questions, different answer scales and different data-gathering methods (e.g., online questionnaires, human-assisted interviews, etc.); (6) many studies do not distinguish between various forms of pornography distribution; (7) available studies are almost exclusively based on declarative data, which is often an inaccurate and biased proxy of actual behavior.

In view of these issues, we conducted an analysis based on objective website traffic data, aimed at estimating the changes in the prevalence of online pornography between 2004 and 2016 among the Polish population. The timespan between 2004 and 2016 is a period of fast proliferation of high-speed broadband Internet technology in Poland, which makes the current analysis even more relevant and interesting. The conducted analysis showed changes in the (1) estimated number of Internet users and (2) online pornography users in the Polish population during the analyzed period. The group of online pornography users was analyzed both as a subset of the general population and as a subset of the Internet user group. Moreover, we analyzed the demographic characteristics (age, sex, population of the place of residence) of pornography users in this period.

Taking the available literature into consideration, our preliminary analysis seems to be the first one aimed at estimating the changes in the prevalence of online pornography use based on objective data and the first study independent of the limitations of the declarative approach discussed in the Introduction. Still, the new methodological path taken in the current study is not devoid of its own pitfalls, which will be elaborated upon in the Method and Discussion sections. The current study was designed to constitute the first step toward supplementing available, declarative data on pornography consumption with objective data, and should inspire further research.

## Method

### Participants and Procedure

Our analysis was based on data that originate from the GemiusAudience panel survey conducted in Poland between the years 2004 and 2016. GemiusAudience is a recognized survey panel that provides data on web traffic for several countries in Europe, and is the biggest panel survey of its kind in Poland. It is also treated as a golden standard for web traffic measurement for major Polish marketing companies and advertisers. The panel study is conducted in accordance with research standards appointed by European Society for Opinion and Marketing Research (ESOMAR, https://audience.gemius.com/en/). In the panel study, participants were enrolled through pop-up questionnaires that were displayed randomly to Internet users visiting popular Polish websites. After giving informed consent to participate in the study and filling out a short questionnaire with their basic demographic data (sex, age, population of the place of residence and others), participants’ Internet activity could be anonymously followed, either using information stored in cookie files or with a special browser add-on. As a reward for participating in the study, all participants took part in periodical prize lotteries—prizes were usually worth around a few hundred zlotys (50–250$). They also had the opportunity to participate in other paid surveys that were conducted among panel study participants. From the viewpoint of research participants, the panel study did not require effort. After the introductory questionnaire, the study was conducted *in the background*, aside from periodical information about prize lotteries and invitations to other paid surveys. Children and adolescents under the age of 18 years old had to have a parent or guardian confirm and consent to their participation in the study. Research participants could resign their participation in the panel study at any moment.

The methodological approach described above allowed us to determine if a particular research participant had visited a specific website, or group of websites (e.g., pornographic websites), during a period of interest. Based on the socio-demographic profiles of participants and their activity on the Internet, it was possible to develop a model reflecting the strength of the relationships between socio-demographic characteristics and the probability of visiting pornographic websites during a particular period of time.

In our analyses, we focused only on visits to websites with pornographic or erotic content. Such websites constitute a category in the panel study with the highest number of websites (other categories were, for example, Education, Sport, Work and Business, Finances and Law, etc.). Webpages were assigned to a specific category after they reached a given number of views by study participants, and were manually reviewed by specialized Gemius workers. In the panel study, pornographic websites included a broad span of Internet sites with pornographic and erotic content: websites with pornographic videos, explicit pictures but also erotic chat groups and other webpages with pornographically themed content (like websites dedicated to pornographic stories). Any dating and sexting sites were, however, outside the scope of this category.

The analyses we conducted were based on the time period between 2004 and 2016. However, the analysis is not based on the whole 12-year period (it was not possible for methodological and financial reasons), but seven separate monthly periods. Specifically, the analysis was based on data reflecting Internet activity in the month of October, for every other year, starting from the year 2004, and ending in 2016. In other words, the analysis included 7 monthly periods: October 2004, 2006, 2008, 2010, 2012, 2014 and 2016. These periods can be compared with each other in terms of each estimated number of online pornography viewers or the proportion of Internet users who viewed pornographic websites. This allowed us to track the time-related shifts in the prevalence of online pornography in the population and among Internet users. The month of October was chosen among other months because of its relative neutrality (month without extended holiday periods, etc.).

During each of the seven monthly periods, the panel study had at least 10,000 participants (specific sample sizes are available in Table S1 on the OSF platform, https://osf.io/v2k5w/). The overall sample size for the panel study was 241,102. Simultaneously, we were provided with data on the socio-demographic structure of Polish Internet users for separate months and weighted statistics for the general population.

The information needed for the weighting and extrapolation procedure came from a parallel representative CAPI study (computer-assisted personal interview; sample *n* = 1700 for every monthly period). The study provided information about Internet usage and socio-demographic characteristics of Polish citizens during a given measurement period (October of a particular year). This data was then used to create weights for panel study participants. Weighting was conducted with respect to three demographic variables used in our analysis: sex, age and population of the place of residence. The weighting procedure was aimed to level potential differences in the socio-demographic structure of panel study participants and the representative group of Internet users in Poland. The number of Internet users and online pornography users in the Polish population (aged 7 years or older) was then estimated based on the weighting procedure.

To sum up, aside from raw data from the study, we were also provided with the weighted and extrapolated data for the general population of Polish Internet users, which reflected the estimated number of Internet users and online pornography users in the Polish population during a particular period. Table S2 available on the OSF platform (https://osf.io/v2k5w/) contains population estimates after weighting and extrapolation procedure, which are the basis for part of the analysis presented in the Results section.

#### Data Constraints

Moreover, the provided panel survey data had 3 methodological constraints, which are worth mentioning upfront:

(1) In the presented analysis, a particular Internet user was classified as a pornography viewer if he or she visited at least one Internet website with pornographic content during the given monthly period. The panel study data did not allow for the analysis of pornography use in terms of its intensity, as the variable reflecting pornography use has a dichotomous character and carries the information only if a particular Internet user visited a pornographic website during the given monthly period or not. (2) The analysis is based only on activities done online with the use of desktops, laptops and notebooks. Internet activity carried out with the use of mobile devices (e.g., smartphones, tablets) is outside the scope of the panel study data and, as a result, our analysis. (3) Additionally, panel study data does not reflect Internet activity carried out using a private browsing mode (or Incognito mode). These issues are further elaborated on in the Discussion section.

### Measures

All demographic variables analyzed in the study were gathered through online pop-up questionnaires, during the sign-up procedure for the study. Participants were asked about their s*ex* (*male* or *female*)—in our analysis, *female* was denoted by 0, while *male* was denoted by 1.

*Age* was measured with the question *What year were you born?* The year of birth reported by participants was then subtracted from the year of the study, and, thus, the participant’s age was calculated. However, in our analysis, *Age* is a categorical variable. Participants were divided into 11 age groups, coded so a higher score reflected older participants: age 7–12 (coded as 1); 13–17 (2); 18–22 (3); 23–27 (4); 28–32 (5); 33–37 (6); 38–42 (7); 43–47 (8); 48–52 (9); 53–57 (10); 58 + (11). The described age categories were proposed by the data provider. Using narrower categories would result in smaller number of research participants in each of them, decreasing the reliability of the results. When discussing the age categories with the data provider, we were especially interested in dividing the categories in such a way that people on both sides of the age of consent (18 y.o. in Poland) would fall into two separate categories, which is indeed the case in the current analysis (we have “12–17 y.o.” and “18–22 y.o.” categories).

*Population of the place of residence* was measured with a single question *How populated is the place you live in?* with 7 answer categories: village (coded as 1), town with a population size of up to 20 thousand, (2) town with a population size between 20 and 50 thousand, (3) town with a population size between 50 to 100 thousand, (4) city with a population size between 100 and 200 thousand, (5) city with a population size between 200 and 500 thousand, (6) city with a population size of over 500 thousand (7). Thus, a higher score indicated that the place of the participant’s residence had a bigger population.

*Period of measurement*, which is also one of the predictors in our analysis, reflects one of the seven monthly periods in which the measurements were taken. As mentioned before, the data reflects online activity during the month of October of every other year, starting from 2004 and ending in 2016.

*Online pornography use* is the dependent variable in our analysis. It is a dichotomous variable that reflects whether a survey participant visited at least one pornographic website during a monthly period in the analysis (1–yes, 0–no).

### Analytic Design

The first part of our analysis is based on investigating the descriptive statistics regarding the prevalence of pornography use (1) in the general population and (2) in the internet user group, depending on the period of measurement and demographic variables. Next, to statistically investigate the effects of demographic variables (age, sex, population of the place of residence) and period of measurement on the probability of pornography viewership among Internet users, we conducted a hierarchical binomial logistic regression analysis. Due to the data structure, we used a multi-level modeling approach where the individuals’ data were nested within periods of measurement (*n* = 7). For the purpose of MLM modeling, all variables were recoded to represent a range of 1, so that the coefficients and odds ratio could be comparable and represent the change in probability as from the lowest value to the highest value of the predictor variable. The period of measurement number was coded as a 0–6 variable where 0 was used for the 1st period of measurement in the study—October 2004.

For our analysis reported here, we decided to use weighted data, to minimize the panel non-representativeness resulting from skewed sampling procedures. We weighted our data at the individual and group (measurement) level. At the individual level, we used ranking weighting (Battaglia et al., 2009; Battaglia et al., 2004) and adjusted data to socio-demographic variables (age, gender, population of the place of residence). At the 2nd level, we adjusted weights for the inequality of sample sizes across measurement periods. A supplementary non-weighted analysis led to similar results and is available on the OSF platform (https://osf.io/v2k5w/). All the variables were group mean centered and orthogonalized. We based our analysis on the bottom approach as suggested by Hox et al. (2017). In this strategy, more complex models are built upon a comparison with simpler models. In the first step, we regressed all the individual level variables on the probability of viewing pornography, along with relevant interactions. In the next step, we introduced the period of measurement as one of the predictors of pornography viewing. In the last step, we introduced interactions between the period of measurement and relevant socio-demographic factors. See the Results section for more details on our analysis.

## Results

### Internet and Online Pornography across Time–Population Estimates

#### Online Pornography Users and Internet Users

**Fig. 1:**
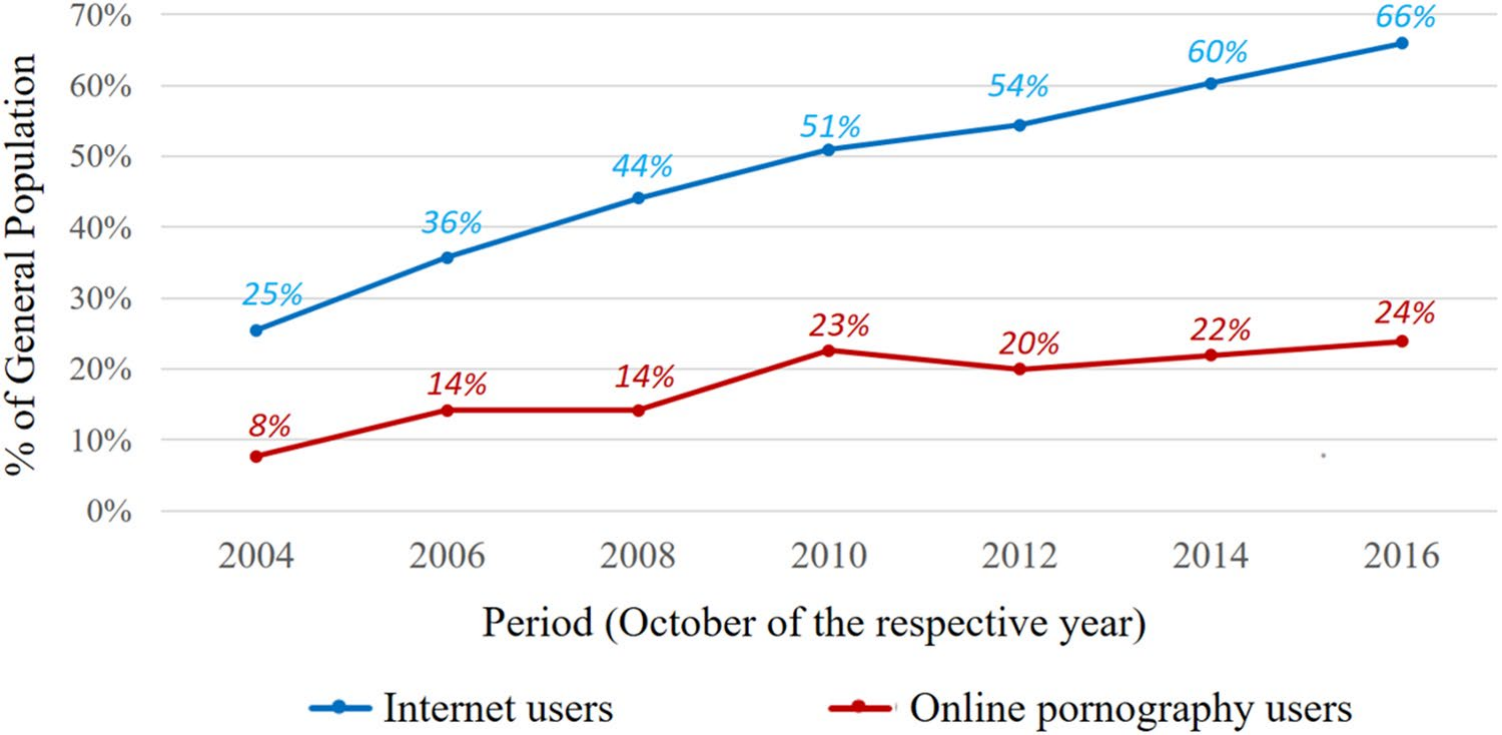
Changes in the estimated numbers of Internet users and online pornography users in Polish population, between years 2004 and 2016 (month October of every other year). General population percentages for the specific periods are given above the respective chart lines

Figure 1 depicts changes in the estimated number of Internet users and online pornography users within the Polish population over consecutive monthly periods between 2004 and 2016. The figure shows what percentage of members in the general population (aged 7 years or older) used the Internet, or Internet pornography, during a particular monthly period. Figure 1 contains visual representation of the data in terms of percentages, whereas exact, absolute numbers are shown in Table S2 in the Supplement.

A broad conclusion that can be drawn from this data is that both the number of online pornography viewers and the number of Internet users generally appeared to increase over consecutive time windows. The estimated number of Internet users rose from just over 9 million users in October of 2004 to over 23.5 million in October of 2016. Within the same time window, the estimated number of pornographic website viewers increased from over 2.75 million to almost 8.55 million (Fig. 1, Table S2). The proportion of people in the general population who used the Internet from their desktop and laptops increased in all successive periods. The pattern is similar—but not the same—for online pornography users. In this case, percentages rose between most successive periods, except for the change between October 2006 and 2008 (the results are stable there, 14.2%) and between October 2010 and 2012 (the percentage dropped from 22.6 to 20.0%). However, the overall trend of the number of people who view online pornography in the population had been unambiguously rising—starting from only 7.7% of the population using online pornography in October 2004 to 25.10% in October 2016. In fact, the relative increase of the number of people using pornography was slightly stronger than the growth of general Internet use. There was an increase of over 310% in the estimated number of Polish population members who used online pornography between October 2004 and October 2016. The Internet user group grew 260% in the same time frame (Fig. 1).

#### Online Pornography Users as a Part of Internet User Group

**Fig. 2:**
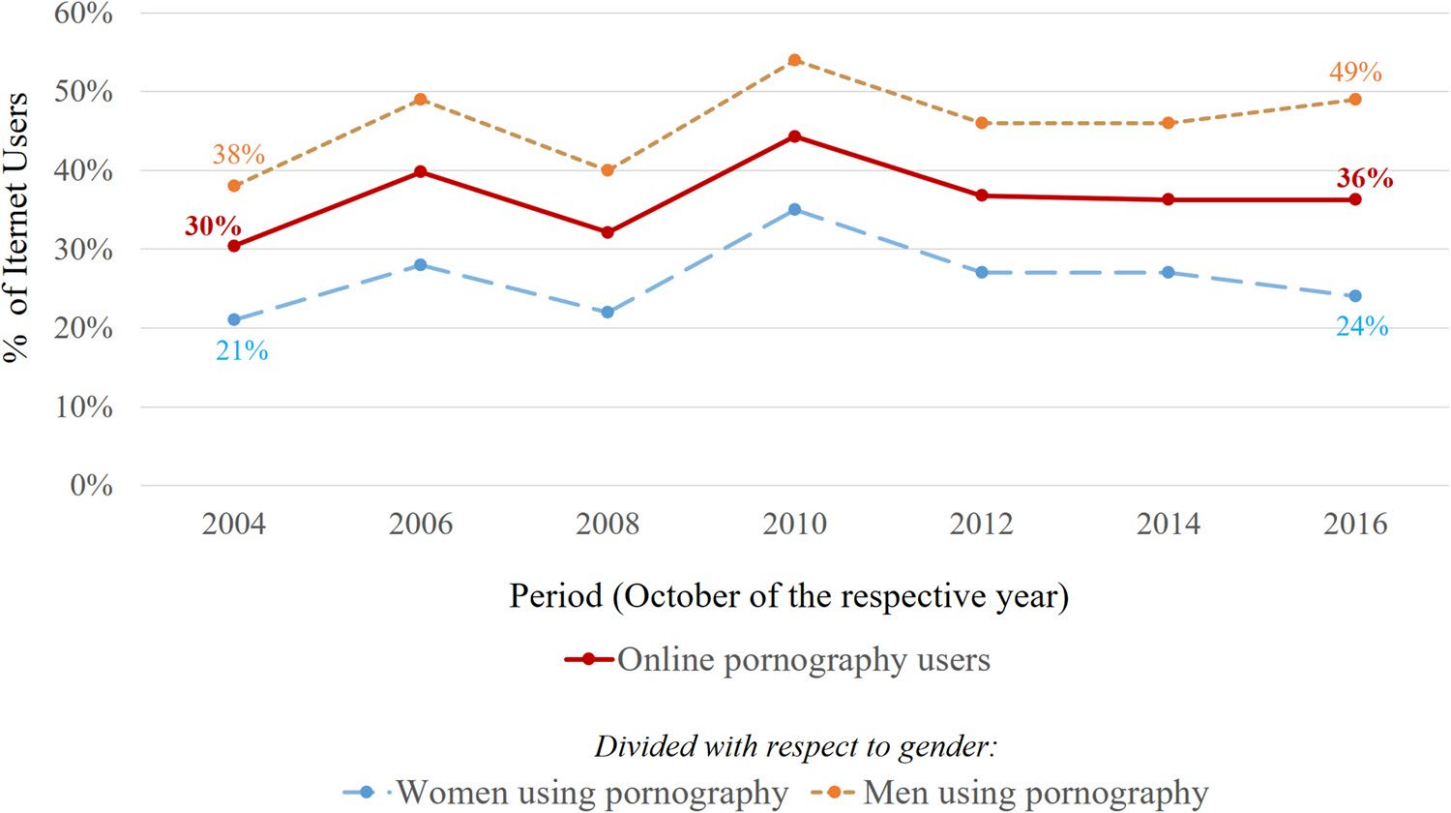
Changes in the proportion of Internet users, who viewed pornography online during a particular monthly period between years 2004 and 2016 (month October of every other year). The chart depicts changes in the percentages for the overall group of online pornography users, as well as the percentages for males and females separately

In Fig. 2, the online pornography user group is presented as a subset of the Internet user group. The data is presented in terms of percentages of Internet users who viewed pornography online. Figure 2 shows the estimated percentages for all users, as well as for females and males separately.

In October 2004, 30% of Internet users also used online pornography. For the corresponding month in 2016, the percentage was higher—36%. Moreover, the increase between the first and the last period of measurement was more visible among males (11% increase, from 38 to 49%) than for females (only 3% increase, from 21 to 24%). Although the trend seems to be slightly upward, the pattern of obtained results is not very straightforward. For both females and males, the proportion of Internet users who used online pornography was lowest for the first period of measurement (October 2004), but we also observed multiple changes in direction of the trend in the first part of the graph. Concurrently, the highest prevalence of online pornography use among Internet users was observed for October 2010 (35% for females and 54% for males). The results were more stable between October of 2012 and 2016. The overall shape of the graph is similar for both males and females, which indicates that a particular pattern of results is not due to gender (Fig. 2).

#### Online Pornography Users–Demographic Characteristics

**Fig. 3:**
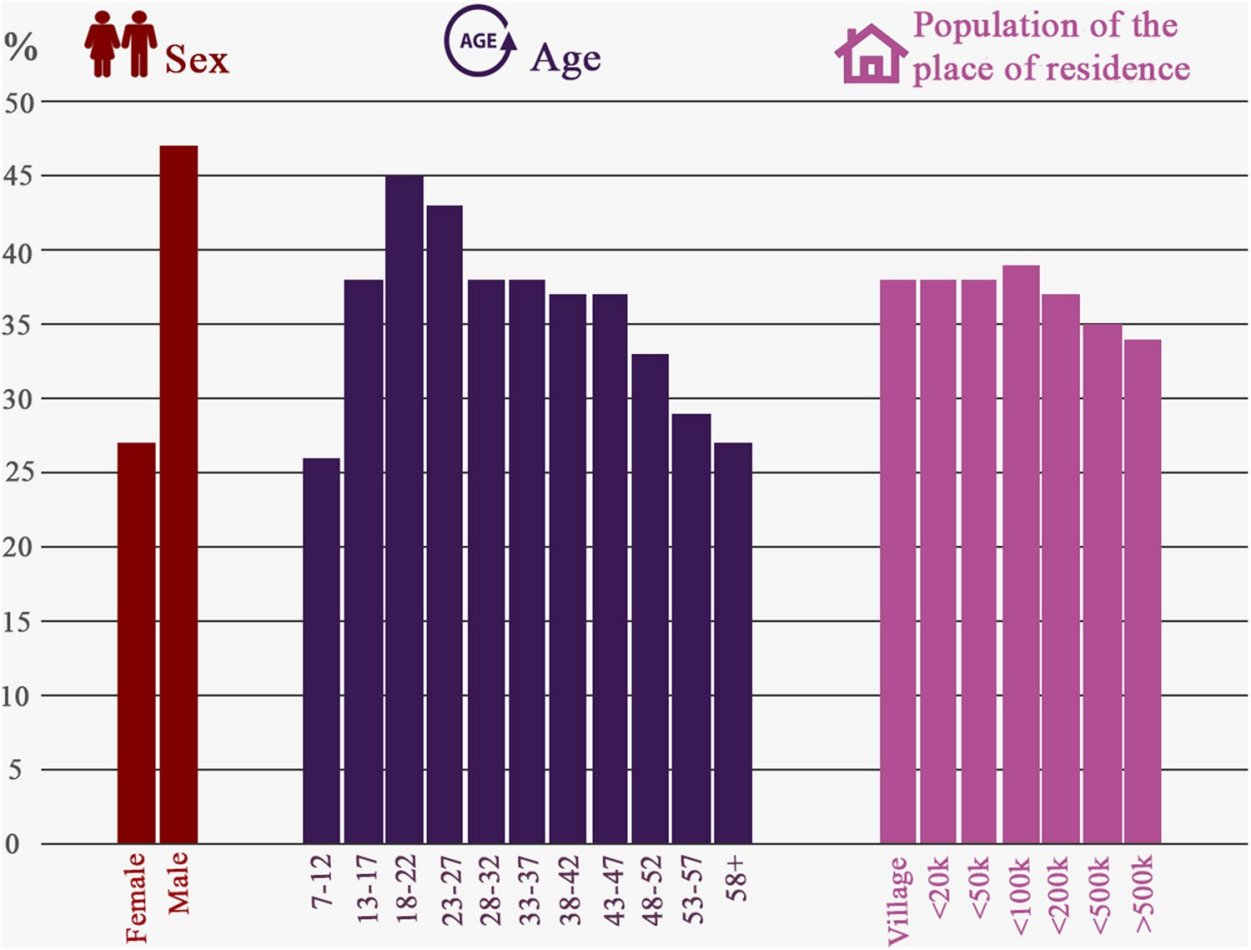
Percentages of Internet users who viewed pornography online, divided with respect to demographic variables: (1) gender, (2) age and (3) population of the place of residence. The charts reflect percentages averaged for all periods of analysis (7 monthly periods, October of every other year, between 2004 and 2016) based on weighted population estimates

Figure 3 depicts percentages of Internet users who viewed pornography online depending on their demographic characteristics: (1) gender, (2) age and (3) population of the place of residence. The chart reflects indices for all analyzed periods taken together (an average from 7 monthly periods of October 2004–2016). The highest difference was noted with respect to gender, with male Internet users having a higher probability to use pornography during a particular month (47%) than female Internet users (27%). In terms of age, the highest proportion of pornography viewers among Internet users was observable for the age groups: 18–22 (45%) and 23–27 (43%). However, prevalence of pornography viewership was also relatively high among younger participants: adolescents (13–17 years, 38%), and even children (7–12 years, 26%). Generally, we noted that at least 25% of Internet users in every age category viewed pornographic websites. Based on the charts, the population of the place of residence did not seem to be as strong a predictor of online pornography use as the previous two variables (Fig. 3).

Table S2 in the Supplement section contains similar data, showing estimated prevalence of pornographic website viewership among Internet users by their demographic characteristics, but presented separately for each of the seven periods of measurement.

**Fig. 4:**
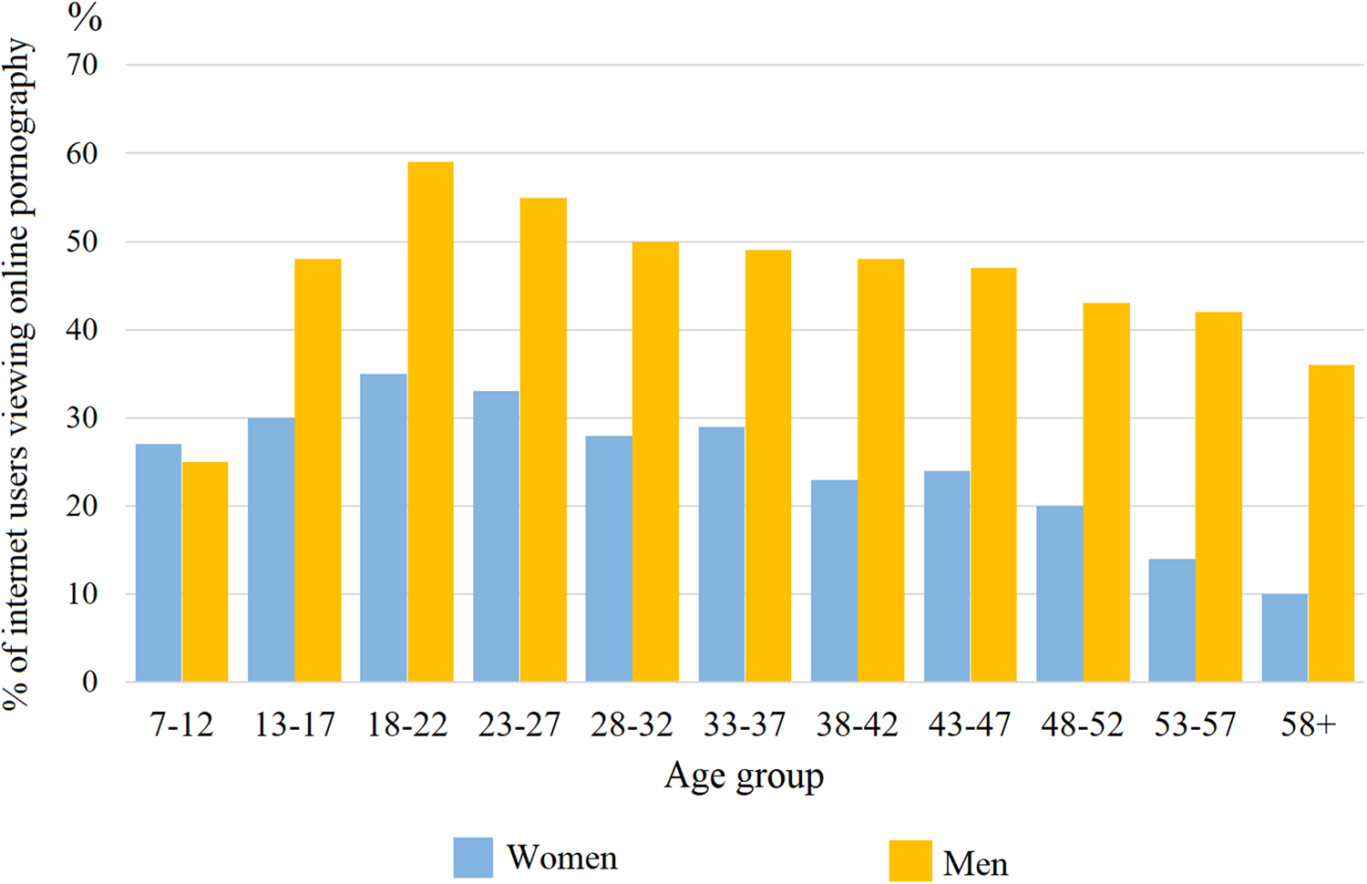
Percentages of Internet users who viewed pornography online, divided with respect to gender and age. The chart reflects percentages averaged for all periods of analysis (7 monthly periods, October of every other year, between 2004 and 2016) based on weighted population estimates

As gender and age seem to have the strongest influence on the probability of viewing online pornography among Internet users, we also investigated the proliferation of pornography viewing depending on these two variables. The cross-tabulation is depicted in Fig. 4. This figure shows the probability of viewing pornographic websites among Internet users with respect to their age and gender (averaged for all measurement periods) (Fig. 4).

Figure 4 shows that the difference between male and female Internet users in terms of the probability of accessing pornographic websites is very strong for each age category, except the youngest Internet users in the 7–12 age span. For this age group, girls who used the Internet had as high a probability of viewing erotic websites (27%) as boys (25%). Moreover, the high probability of viewing online pornography seems to be maintained for longer among males than among females, as the highest difference between genders was obtained for the 53–57 age group (14% of female and 42% of male Internet users viewed pornographic websites in this age group) and the 58 + age group (10% of females, 36% of males).

### Logistic Regression Analysis Predicting Pornography Viewership among Internet Users

We conducted a hierarchical binomial logistic regression analysis to investigate the effects of demographic variables (age, sex, population of the place of residence) and period of measurement on the probability of pornography viewership among Internet users (Table 1). As mentioned in the Data analysis subsection, we based our analysis on the bottom approach as suggested by Hox and colleagues (2017). The Inter Class Correlations for the initial empty model was 0.01, meaning that pornography viewership was localized mainly at the individual level. In Step 1, we regressed all the individual level variables on the prevalence of viewing pornography among Internet users and additionally added two individual level interactions between (1) gender and age, (2) gender and age squared (see Table 1). The analysis showed that adding the individual interactions significantly improved the predictive power of the model. In Step 2, we checked whether adding the period of measurement as a predictor for using online pornography significantly would improve the predictive power and the global fit to the data. Next, in Step 3, we investigated whether relations between individual level predictions vary significantly between different periods of measurement. And finally, in Step 4, we analyzed whether variation in slopes may be explained by the period of measurement. The analysis showed that the model that fitted the data most appropriately contained only 1st level individual predictors while allowing for the variance of slopes across the measurement points—a random slope model. Introducing the period of measurement as a predictor did not significantly increase the predictive power of the model based on the log-likelihood criterion (detailed results of model comparisons are available on the OSF platform, https://osf.io/v2k5w/). To clearly illustrate the dependencies between the influence of socio-demographic factors and the period of measurement on the prevalence of pornography viewing (which were also discussed in the earlier, descriptive part of our analysis; Figs. 2 and 4), we decided to present three parallel models (Table 1).

**Table 1:** Results of multi-level logistic regression for the pornography usage-weighted estimation

	Model 1				Model 2				Model 3			
	*OR (CI)*	*B*	*SE*	*var*^*rs*^	*OR (CI)*	*B*	*SE*	*var*^*rs*^	*OR (CI)*	*B*	*SE*	*var*^*rs*^
Period	-	-	-	-	0.92 (0.88, 0.97)**	− 0.08	0.02	-	0.93 (0.89, 0.99)*	− 0.06	0.02	-
Sex^b^	2.60 (2.36, 2.86)***	0.95	0.04	.01***	2.60 (2.36, 2.85) ***	.095	0.04	.01***	2.50 (2.19, 2.86)***	0.92	0.05	.01***
Age	0.34 (0.24, 0.48)***	− 1.08	0.14	0.10***	0.34 (0.24, 0.48)***	− 1.08	0.15	0.10***	0.19 (0.15, 0.27)***	− 1.61	0.12	0.01**
Age^2^	0.48 (0.34, 0.68)*	− 0.74	0.14	0.08***	0.47 (0.33, 0.67)**	− 0.75	0.14	0.08***	0.44 (0.27, 0.72)**	− 0.81	0.19	0.10***
Residence	0.88 (0.77, 1.02)	− 0.12	0.06	0.01***	0.86 (0.77, 1.01)	− 0.12	0.06	0.01***	0.89 (0.77, 1.02)	− 0.12	0.06	0.02***
Sex x Age	2.39 (1.68, 3.40)**	0.87	0.14	0.07***	2.39 (1.67, 3.43)**	0.87	0.15	0.07***	2.41 (1.68, 3.45)***	0.88	0.15	0.07***
Sex x Age^2^	0.88 (0.66, 1.21)	− 0.12	0.12	0.04***	0.89 (0.66, 1.22)	− 0.11	0.13	0.04***	0.91 (0.66, 1.25)	− 0.12	0.13	0.05***
Period x Sex ^a c^									1.01 (0.98, 1.04)	0.01	0.01	-
Period x Age ^a c^									1.20 (1.08, 1.33)**	0.18	0.04	-
Period x Age^2 a c^									1.02 (0.91, 1.14)	0.02	0.06	-
*N1*/*N2*									210448/7			

*p < .05; **p < .01; ***p < .001

^a^–Period of measurement number was coded as 0–6 variable where 0 was used for 1st period of measurement in the study—October 2004; ^b^ gender was coded as coded as 0 for women and 1 for men; ^c^ the analysis shows whether the relation between pornography watching and specific predictor increases or decreases linearly across measurement times; ^rs^–variance component for random slopes, significance means that there is a significant variance between measurement periods

In Model 1, we regressed sex, age, population of the place of residence and measurement period on the probability of viewing pornography among Internet users. For age, we included both linear and quadric terms to reflect the nonlinear relationship between age and online pornography viewing. We also included interaction terms for 1st level interactions between sex and both age terms. In Model 2 and 3, we extended our basic model by adding interaction terms between period of measurement and sex and period of measurement and both age terms (Table 1). We did not include interactions for the population of the place of residence because (1) this variable has less theoretical importance for pornography viewing than sex and age; (2) as a result of the inspection of descriptive statistics (presented in the earlier parts of the results section); (3) as well as the fact that this variable did not have a significant main effect on the prevalence of pornography viewing.

The obtained results showed that online pornography viewership was more prevalent among male Internet users than among female users. In Model 1, both linear and quadratic age effects were significant and negative. These two effects reflect the curvilinear relationship between age and probability of viewing online pornography among Internet users (the probability is lowest for youngest and oldest Internet users, and higher in the middle of the age distribution). For Models 2 and 3, the effect of period of measurement on pornography usage (monthly periods, October every other year between 2004 and 2016) was marginally significant and negative, but adding it into the models did not improve the model fits, which is in agreement with the weighted population estimates presented earlier in Fig. 2. The population of the place of residence did not seem to significantly influence the probability of viewing online pornography among Internet users in the basic, or in the extended regression model. The only 2nd level interaction included in the extended regression models that was significant was the interaction between measurement period and age, but its role in explaining online pornography viewership among Internet users was rather small. Adding all the 2nd level interactions into models did not improve the global fit of the models.

## Discussion

Our results indicated that the estimated proportion of the population that used online pornography decidedly increased in the period between October 2004 and October 2016. Based on our data, there was over a threefold increase (310%) in the proportion of population members who used online pornography–starting from an estimated value of 7.7% in October 2004 to 24% in October 2016. On the level of estimated absolute values, the number of population members who viewed pornographic websites in October 2004 was 2.76 million, and grew to 8.54 million in October 2016—this constitutes a difference of almost 6 million people (Fig. 1, Table S2). When it comes to Internet users, the percentage of people using pornography in this group did not seem to change as significantly (Table 1), which will be discussed in the further sections.

The growth trend in the prevalence of online pornography seems to be propelled mostly by the expanding reach of the Internet during this period in Poland. The corresponding, relative increase in the estimated number of Internet users was similar in pattern, but only slightly smaller than the increase for online pornography. The estimated proportion of Internet users in the general population increased 2.5 times in the analyzed period–from 25% (estimated 9.1 million) to 66% (23.5 million).

The increase in the indices of online pornography prevalence based on our data is decidedly faster than the similar trend observed in the data gathered through the General Social Survey, which was described in the Introduction section (Price et al., 2016; Wright, 2013). This is not surprising, as some researchers suggest that the change in the prevalence of pornography reflected in the GSS data is slower than expected (e.g., Wright, 2013). However, it is important to note that there are important differences between the GSS and our analysis.

Our analysis is focused on only one form of pornography distribution, that is—online pornography. In contrast, the GSS data may also reflect other forms of access to pornography. As mentioned before, the label “X-rated movie” used in the GSS may also correspond to pornography bought or rented on VHS/CD/DVDs. Indices based on the GSS data may maintain relative stability over time because they reflect two latent components–the growth trend in the prevalence of online pornography, and the decreasing popularity of more traditional forms of pornography distribution.

Although there is not much data available on this topic, market analyses seem to confirm the described shift between popular mediums of pornography distribution. In the USA, the profits from pornographic video sales and rentals started to fall sharply in the mid-2000s. Compared to the year 2005, the profits noted for 2006 were smaller by 15.4%. In the same time frame, the profits from online pornography rose by 13.6% (Edelman, 2009). This shift is convergent with the creation of so-called tube-porn sites, which is discussed below.

The downward trend of pornographic video sales likely continued in the successive years. The Free Speech Coalition, the trade association of the pornography and adult entertainment industry in the USA estimated that profits from this branch of pornography distribution decreased by 50% between 2007 and 2011 (Barrett, 2012).

On the basis of these reports, it is likely that the first decade of the 2000s marked a dramatic change in the way that most people accessed pornography—from pornographic magazines and rental movies, to streaming pornographic content online. Our data is convergent with this notion, as it reflects the growing popularity of the Internet and online pornography in this period. The analysis based on GSS data may be blind to such changes, as in the GSS, the domain of pornography distribution is not specified.

Although our analysis indicates a clear increase in the prevalence of pornography in the population, it is not as clear whether online pornography also became increasingly popular within the existing Internet user group. Our results indicate that an estimated 30% of Internet users viewed pornographic websites in October of 2004, while 36% did so in October 2016. Interestingly, the growth in the prevalence of online pornography among Internet users is more visible among men, than among women. For men, we noted an 11% increase between the first and the last period of measurement (38% for October 2004, 49% for October 2016), while for women, it was only 3% (21–24%). It is possible that the higher availability of pornography had more influence on male Internet users than on female users because of a higher sexual drive and a higher need for sexual stimulation among males (Baumeister et al., 2001).

While comparing only the first and the last period of measurement indicated higher estimated proliferation of online pornography among Internet users, we also noted multiple changes in the direction of the trend in the intervening periods. The most visible changes were noted between 2006 and 2010: 40% of Internet users viewed online pornography in October 2006, 32% in October 2008 and 44% in October 2010. Because of these changes, the effect of the period of measurement on the probability of watching pornography did not provide a significant added value to our regression models (Table 1).

Firstly, it is important to consider the cultural context of the findings, as it may influence how our results would translate to other populations. The analysis is based on Polish participants. Poland is among the most religious countries in the European Union, with a dominant Catholic affiliation (Eurobarometer, 2019). This is important, as religiosity can be related to moral disapproval of pornography and can influence pornography viewing frequency (Grubbs et al., 2019a, 2019b). Recent studies conducted on representative populations of the USA (Grubbs et al., 2019a, 2019b) and Poland (Lewczuk et al., 2020; Lewczuk et al., 2021) showed that the level of moral disapproval of pornography was very similar in both countries (only slightly less in the latter). Moreover, US data shows a growing acceptance of pornography through the years (Gallup, 2018; Lykke & Cohen, 2015). Unfortunately, similar data for the Polish population is not available but if a similar pattern is true, it would constitute another factor possibly contributing to the growing rates of pornography consumption reported in the current analysis. Poland is an ethnically homogeneous country, with a predominant Caucasian ethnicity. As sexual behavior and pornography use patterns can differ depending on factors like race (e.g., Perry & Schleifer, 2018), results of similar analyses in ethnically and racially differing communities could be different.

Additionally, although this argument is speculative in nature, we suggest that the prevalence of online pornography among Internet users may be the most alternating due to 3 factors, delineated below.

**Fig. 5:**
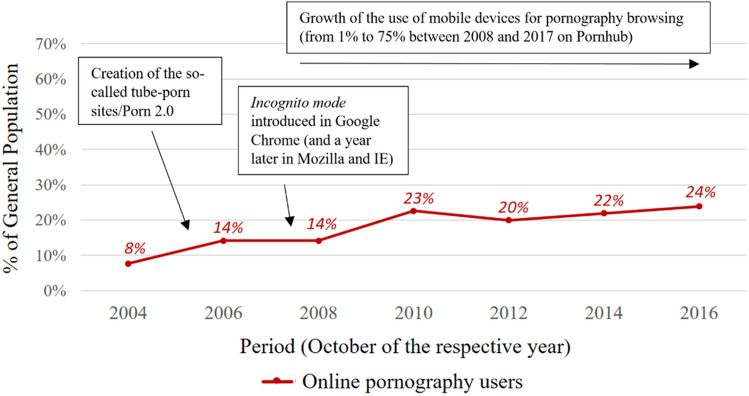
Chart depicting changes in the estimated numbers of online pornography users in Polish population, between years 2004 and 2016, with the most important changes in pornography delivery technology marked on the graph. General population percentages for the specific periods are given above the chart line

(1) Starting in 2006, so-called tube-porn sites appeared on the Internet (also referred to as “Porn 2.0,” which is a reference to the Web 1.0 and Web 2.0 distinction) (Mowlabocus, 2010; Tyson et al., 2013). These kinds of sites, modeled on the YouTube platform, allowed for free, on-demand streaming of pornographic materials. Similar to YouTube, the content could be uploaded by users and social features were also implemented: user ratings, video comments and communication between users. Such a design promoted user engagement, instant gratification and allowed users to view many pornographic clips in a relatively short time. These features were likely intended to attract new users of online pornography. One can speculate that this may be at least partly connected to the increase in the prevalence of pornography viewership among Internet users between October 2004 and October 2006 observed in our data.

(2) Private browsing modes allow users to browse Internet websites without leaving a trace on the local disk of a computer, i.e., without storing information in the browser history, or in the browser cookie files (e.g., Aggarwal et al., 2010; Gao et al., 2014). Because the panel study that our data originates from was based on gathering information stored in cookie files, the presented analysis excludes web traffic generated while using a private browsing mode. Private browsing mode was first introduced in 2005 for the Safari browser, then in 2008–09 for Windows browsers. This can potentially be one of the potential reasons for the obtained decrease in indices between 2006 and 2008, although this explanation must be confirmed in future studies. Since browser producers do not share or publish this kind of data, it is hard to reliably estimate what portion of visits on pornographic sites is accessed through private browsing presently, and before private browsing gained popularity. Aggarwal and colleagues (2010) showed that private browsing is used more frequently on adult sites than on news sites or for online shopping. However, it is also possible that growing acceptance of pornography (Gallup, 2018; Lykke & Cohen, 2015) can result in fewer people using private browsing to “hide” their pornography consumption.

(3) The other factor that could have a strong influence on the observed prevalence of online pornography use among Internet users is the outflow of pornographic website views from desktops and laptops to mobile devices (e.g., smartphones, tablets and palmtops). As our analysis is based on web traffic data generated from desktops and laptops, it is highly possible that it significantly underestimates the prevalence of online pornography use among both Internet users and in the general population. According to the statistical report published by one of the biggest pornographic websites (Pornhub, 2018), the number of website views via mobile devices, irrespective of the country, may currently exceed views generated on desktops and laptops. According to the statistical report provided to us by Gemius (private correspondence), our panel survey data provider, for October 2016, aside from an estimated 8.54 million Polish users viewing pornography on their desktops and laptops, 5.48 million users viewed pornography using their mobile devices. Summing up both of these indices, it is estimated that 39% of Polish population members used pornographic websites in October 2016. The data encompassing mobile devices were available only for October 2016 and due to this are not a part of our statistical analysis, being presented here as an additional point. To accurately establish the prevalence of pornography use in populations, we need further research that would allow us to investigate the prevalence of pornography use on both mobile devices and desktops or laptops. One other very interesting research direction that has not been initiated is on the differential effects (positive or negative) of viewing pornography on mobile devices, as compared to desktops and laptops. Does using pornography on a personal smartphone have different characteristics than viewing pornography on other devices? This topic is especially important as pornography consumption through mobile devices will likely be even more prevalent in the future. Figure 5 depicts the changes that had an important potential influence on the way pornography was proliferated during the analyzed period as previously discussed.

Although the factors presented above can be important, and are surely interesting in light of our findings, it is important to underline once again that the points discussed above are of a speculative nature and we cannot prove their importance based on our data alone.

Summing up, the increase in the prevalence of online pornography in the timespan between 2004 and 2016 is evident based on our data. Because of the limitations of the current analysis mentioned above, and because of problems inherent to assessing the prevalence of pornography use with declarative measures, more studies based on objective data (e.g., website traffic data) are needed. Future studies should provide the necessary context for the results we are reporting.

Aside from the analysis of the prevalence of online pornography viewership in the general population and among Internet users, we also conducted an analysis of the influence of demographic characteristics (sex, age, population of the place of residence) and period of measurement, on the probability of viewing Internet pornography among Internet users. Said analysis showed that males using the Internet were much more likely to use Internet pornography than females (47% vs. 27%). This particular result is within the range of results usually obtained from declarative data (Grubbs et al., 2019a, 2019b; Short et al., 2012; Wright, 2013). Additionally, our analysis showed that the probability of viewing Internet pornography among males is decidedly higher than among females for every age category, except for the youngest category, between 7 and 12 years old. For this age category, 27% of girls used the Internet, and 25% of boys viewed pornographic websites (Fig. 4). At first glance, this result can seem paradoxical; however, it is in line with the results of recent surveys, according to which the age of first contact with pornography is very similar for boys and girls (Opinium Research, 2015). It is important to notice that the first visits on Internet websites with pornographic content are likely driven by curiosity, and not factors directly related to sexual drive. It is still possible that among Internet users aged 7–12 boys view pornography with higher frequency than girls, as our data does not allow for analysis of pornography consumption in terms of its intensity (a particular person was classified as viewing pornography if he/she visited a pornographic website at least one time during a particular monthly period). However, for the other age groups we noted a stark difference in the probability of viewing pornographic websites among males and females. This is also in line with the available literature and can be connected with a higher level of sexual drive among males, which starts to appear in adolescence (Baumeister et al., 2001).

The analysis we conducted also showed the curvilinear relationship between age and probability of viewing pornography among Internet users. Our results indicated that starting from the first age category (7–12 years) the prevalence of viewing online pornography increased for each successive age category, peaking for individuals who had recently came of age (18–22 years). For subsequent age categories, the trend changed direction and the probability of viewing pornography gradually decreased, although for people aged between 33 to 47 years of age the indices were relatively stable, with a more sudden drop-off after this period (Fig. 3). It is worth noting that, in every age category, there was at least 25% of Internet users who used pornography, including the youngest (7–12) and oldest (58 +) age group. As some researchers suggest, it is possible that online pornography democratized pornography access, and made it more accessible especially for demographic groups among which pornography consumption through traditional media was not usually high (Owens et al., 2012). Nevertheless, the current investigation should be considered preliminary and the results should be replicated in future research.

The analysis we are presenting provides the first available results describing and confirming the increase in the prevalence of online pornography associated with the development of the Internet. We described the results on two levels: prevalence of online pornography in the general population, and among Internet users. For the latter group, we also analyzed the demographic characteristics of online pornography users.

### Limitations

As elaborated above, as well as in the data constraints subsection, our methodological approach also has several important limitations: the panel study data that is the basis of our analysis does not include (1) Internet activity generated while using a private browsing mode (2) and using mobile devices. Moreover, (3) our dataset does not allow for the analysis of prevalence of pornography in terms of the intensity of use. Moreover, the current analysis is based on a heterogeneous group of pornographic and erotic websites (including erotic chat groups, websites with pornographic videos, pictures and stories). Unfortunately, we could not offer a more specific breakdown of visits on pornographic websites. Also, inclusion of a particular website in the category of pornographic services relied on the decision of the company’s employees to identify it as such. This is a constraint, as people can differ in what they regard as pornography (Willoughby & Busby, 2016). The study itself was conducted by an external operator, which means that we did not have control over the design of the study and data-gathering process. While this is surely a limitation of our analysis, it is also true that the provided dataset was quite unique in terms of its characteristics and the analytical opportunities it provided. We are not aware of another source of data that would allow for retrospective assessment of the proliferation of online pornography over the past 12 years based on objective indicators. Moreover, although we discuss several external factors that could influence the shape of the obtained results, we also want to fully acknowledge that other factors not discussed here may also have had an important effect on the pornography proliferation. Lastly, the period of measurement indicator used in our analysis had only 7 levels, which is a relatively small number when our MLM analysis is considered (in which the period of measurement is used as one of the predictors of probability of pornography viewing). To facilitate the process of drawing reliable conclusions from the data, future studies should investigate the subject based on a higher number of analyzed periods. Due to these limitations, as well as the novelty of the methodological approach, the analysis reported here should be considered preliminary and interpreted with caution. An accurate estimation of pornography use proliferation is not only meaningful for purely epidemiological reasons. As frequency of pornography use is an important factor in the leading theoretical models of problematic pornography use and compulsive sexual behavior (Grubbs et al., 2019a, 2019b; Lewczuk et al., 2020), accurate estimation of pornography use can help further research on this subject. Recently, more and more research has shown the subjectivity in viewing own problematic pornography use and pornography viewing habits (Brand et al., 2019; Grubbs et al., 2019a, 2019b). Using objective means to estimate pornography consumption can help to differentiate between actual behavior and subjective appraisals of the behavior, which would be an important factor contributing to the development of pornography research. Moreover, studies on the positive and negative consequences of pornography also have to be supplemented by accurate accounts of (changes in) pornography proliferation, as the effects of pornography are likely to scale with changing proliferation.

Considering the limitations inherent in self-reported data, especially in the domain of sexual behavior, the presented analysis should constitute a first, preliminary step in a series of future studies that aim to estimate the prevalence of pornography use in populations based on objective data.

## Supplementary Information

Below is the link to the electronic supplementary material.

Supplementary file1 (DOCX 21 kb)
