# Supplementary material for: Increase in the Prevalence of Online Pornography Use: Objective Data Analysis from the Period Between 2004 and 2016 in Poland
Source: Arch Sex Behav. 2021 Nov 8;51(2):1157–71. doi: 10.1007/s10508-021-02090-w (PMC8888374; doi:10.1007/s10508-021-02090-w)
Supplement: Supplementary file 1 — Supplementary file1 (DOCX 21 kb) [file 10508_2021_2090_MOESM1_ESM.docx]

**Supplement**

Table S1

|  | Period of the analysis (month October of the respective year) | | | | | | |
| --- | --- | --- | --- | --- | --- | --- | --- |
|  | *2004* | *2006* | *2008* | *2010* | *2012* | *2014* | *2016** |
| *N* | 16 883 | 20 143 | 16 647 | 15 087 | 11 425 | 11 543 | 118 721 |

*Number of panel study participants in each of the seven monthly periods of analysis.*

**the number of participants for the year 2016 is bigger, as the procedure for signing up for the study was simplified for this period (compared to previous ones)*

Table S2

*Estimated numbers of polish population members who used Internet (Internet) or online pornography (Pornography) in each of the seven analyzed periods, divided with respect to sociodemographic characteristics (data after weighting and extrapolation procedure).*

|  |  |  | Period of analysis (month October of the respective year) | | | | | | |
| --- | --- | --- | --- | --- | --- | --- | --- | --- | --- |
|  | | | *2004* | *2006* | *2008* | *2010* | *2012* | *2014* | *2016* |
| Sex | Females | Internet | 4103600 | 6181362 | 7718040 | 9056237 | 9806949 | 10855380 | 11785742 |
|  |  | Pornography | 880844 | 1874675 | 1859444 | 3180250 | 2673021 | 2936195 | 2817662 |
|  |  | % | **21.5%** | **30.3%** | **24.1%** | **35.1%** | **27.3%** | **27.0%** | **23.9%** |
|  | Males | Internet | 4959206 | 6522940 | 7949314 | 9119417 | 9611258 | 10651007 | 11754519 |
|  |  | Pornography | 1877528 | 3176309 | 3173966 | 4879381 | 4464147 | 4873209 | 5726856 |
|  |  | % | **37.9%** | **48.7%** | **39.9%** | **53.5%** | **46.4%** | **45.8%** | **48.7%** |
| Age | 7-12 | Internet | 558707 | 681196 | 1456278 | 1746554 | 2063870 | 1935404 | 808506 |
|  |  | Pornography | 92153 | 183174 | 394018 | 466542 | 544293 | 504889 | 210129 |
|  |  | % | **16.49%** | **26.89%** | **27.06%** | **26.71%** | **26.37%** | **26.09%** | **25.99%** |
|  | 13-17 | Internet | 1282764 | 1369644 | 1537219 | 1741926 | 1419758 | 1786267 | 1774773 |
|  |  | Pornography | 421704 | 618308 | 561922 | 831731 | 437988 | 675895 | 568727 |
|  |  | % | **32.9%** | **45.1%** | **36.6%** | **47.7%** | **30.8%** | **37.8%** | **32.0%** |
|  | 18-22 | Internet | 2568329 | 2939133 | 2418564 | 2679444 | 2424540 | 2172045 | 2028359 |
|  |  | Pornography | 1026436 | 1430720 | 994176 | 1451071 | 1160451 | 954209 | 769558 |
|  |  | % | **39.97%** | **48.68%** | **41.11%** | **54.16%** | **47.86%** | **43.93%** | **37.94%** |
|  | 23-27 | Internet | 1303244 | 1603375 | 2067111 | 2344428 | 2575101 | 2526468 | 2713726 |
|  |  | Pornography | 421154 | 673340 | 753676 | 1239591 | 1174700 | 1132454 | 1071236 |
|  |  | % | **32.3%** | **42.0%** | **36.5%** | **52.9%** | **45.6%** | **44.8%** | **39.5%** |
|  | 28-32 | Internet | 776060 | 1448735 | 1821123 | 2124976 | 2396134 | 2761838 | 2785759 |
|  |  | Pornography | 191734 | 583115 | 629144 | 940661 | 848414 | 1150773 | 1072651 |
|  |  | % | **24.7%** | **40.2%** | **34.5%** | **44.3%** | **35.4%** | **41.7%** | **38.5%** |
|  | 33-37 | Internet | 607293 | 1111151 | 1580551 | 1959205 | 2085774 | 2520429 | 2462092 |
|  |  | Pornography | 163203 | 418303 | 503594 | 879957 | 859805 | 967026 | 946937 |
|  |  | % | **26.9%** | **37.6%** | **31.9%** | **44.9%** | **41.2%** | **38.4%** | **38.5%** |
|  | 38-42 | Internet | 632991 | 935776 | 1345796 | 1357317 | 1636561 | 1976554 | 2410302 |
|  |  | Pornography | 151343 | 367320 | 394845 | 584668 | 622076 | 725625 | 906997 |
|  |  | % | **23.9%** | **39.3%** | **29.3%** | **43.1%** | **38.0%** | **36.7%** | **37.6%** |
|  | 43-47 | Internet | 501651 | 788446 | 1007675 | 1122464 | 1283181 | 1445178 | 2144127 |
|  |  | Pornography | 136193 | 303798 | 272681 | 487162 | 498066 | 511385 | 833011 |
|  |  | % | **27.1%** | **38.5%** | **27.1%** | **43.4%** | **38.8%** | **35.4%** | **38.9%** |
|  | 48-52 | Internet | 382850 | 837798 | 1010973 | 1042236 | 972383 | 1120024 | 1889433 |
|  |  | Pornography | 69587 | 232306 | 218822 | 416593 | 345703 | 350873 | 754543 |
|  |  | % | **18.2%** | **27.7%** | **21.6%** | **40.0%** | **35.6%** | **31.3%** | **39.9%** |
|  | 53-57 | Internet | 260976 | 526424 | 785021 | 1006529 | 1272438 | 1294748 | 1504524 |
|  |  | Pornography | 50953 | 125528 | 181656 | 404326 | 296912 | 359096 | 529342 |
|  |  | % | **19.5%** | **23.8%** | **23.1%** | **40.2%** | **23.3%** | **27.7%** | **35.2%** |
|  | 58+ | Internet | 187941 | 462624 | 637043 | 1050575 | 1288467 | 1967432 | 3018660 |
|  |  | Pornography | 33912 | 115072 | 128876 | 357329 | 348760 | 477179 | 881387 |
|  |  | % | **18.0%** | **24.9%** | **20.2%** | **34.0%** | **27.1%** | **24.3%** | **29.2%** |
| Population of the place of residence | Village | Internet | 1640031 | 3094919 | 4399410 | 5682554 | 6510823 | 7798013 | 8760743 |
|  |  | Pornography | 456698 | 1237668 | 1483350 | 2559965 | 2479973 | 2952941 | 3175846 |
|  |  | % | **27.8%** | **40.0%** | **33.7%** | **45.0%** | **38.1%** | **37.9%** | **36.3%** |
|  | < 20 000 | Internet | 2372939 | 1799092 | 2123713 | 2438181 | 2596117 | 2862865 | 2997718 |
|  |  | Pornography | 594039 | 826888 | 730049 | 1203387 | 1016540 | 1027758 | 1088385 |
|  |  | % | **25.0%** | **46.0%** | **34.4%** | **49.4%** | **39.2%** | **35.9%** | **36.3%** |
|  | < 50 000 | Internet | 1130494 | 1581626 | 1815901 | 1986883 | 2047845 | 2895080 | 2947215 |
|  |  | Pornography | 363174 | 602335 | 603310 | 866465 | 796797 | 1071661 | 1129063 |
|  |  | % | **32.1%** | **38.1%** | **33.2%** | **43.6%** | **38.9%** | **37.0%** | **38.3%** |
|  | <100 000 | Internet | 1045024 | 1320221 | 1577856 | 1723143 | 1774014 | 2045324 | 2239893 |
|  |  | Pornography | 394550 | 553212 | 613540 | 753541 | 719825 | 728627 | 845159 |
|  |  | % | **37.8%** | **41.9%** | **38.9%** | **43.7%** | **40.6%** | **35.6%** | **37.7%** |
|  | < 200 000 | Internet | 911590 | 1337603 | 1391281 | 1655293 | 1772134 | 1484860 | 2005896 |
|  |  | Pornography | 318236 | 536249 | 399292 | 752670 | 651137 | 558532 | 743914 |
|  |  | % | **34.9%** | **40.1%** | **28.7%** | **45.5%** | **36.7%** | **37.6%** | **37.1%** |
|  | < 500 000 | Internet | 828267 | 1520370 | 1796333 | 2102918 | 2036371 | 1824540 | 1953167 |
|  |  | Pornography | 267036 | 592490 | 541921 | 879278 | 560593 | 616446 | 743391 |
|  |  | % | **32.2%** | **39.0%** | **30.2%** | **41.8%** | **27.5%** | **33.8%** | **38.1%** |
|  | > 500 000 | Internet | 1134461 | 2050471 | 2562860 | 2586682 | 2680903 | 2595705 | 2635629 |
|  |  | Pornography | 364639 | 702142 | 661948 | 1044325 | 912303 | 853439 | 818760 |
|  |  | % | **32.1%** | **34.2%** | **25.8%** | **40.4%** | **34.0%** | **32.9%** | **31.1%** |
